# Efficacy of Dexamethasone in Reducing Postoperative Symptoms of the Surgical Extraction of Impacted Third Molars

**DOI:** 10.7759/cureus.72035

**Published:** 2024-10-21

**Authors:** Sangita Kalita, Rohit Goyal, Hem Kumar, Divya Yadav, Lakhan Talreja, Pooja Jaiswal

**Affiliations:** 1 Department of Oral and Maxillofacial Surgery, Maharaja Ganga Singh Dental College and Research Centre, Sri Ganganagar, IND; 2 Department of Oral and Maxillofacial Surgery, Surendera Dental College and Research Institute, Sri Ganganagar, IND

**Keywords:** dexamethasone, impaction, mouth opening, pain, swelling, third molar

## Abstract

Introduction: Among anti-inflammatory medications, dexamethasone has an extended half-life and strong effects, particularly in oral surgical procedures. Administering dexamethasone helps reduce discomfort and inflammation and enhances recovery by minimizing postoperative complications. This study aimed to assess the effectiveness of dexamethasone administered before and after surgery in reducing postoperative pain and swelling and relieving mouth opening following the surgical removal of impacted third molars.

Material and methods: An observational, prospective cohort study was conducted on 60 patients who had already been administered with dexamethasone pre- and postoperatively and were divided into two non-randomized groups. The first group had received 8 mg of dexamethasone intramuscularly one hour before the surgical removal of impacted third molars, while the second group had received the same dose immediately after surgery. The observer evaluated pain, swelling, and mouth opening in both groups at baseline (preoperative) and on the second and seventh days after the surgical extraction of impacted third molars.

Results: In terms of swelling, the second postoperative day showed a significant difference between the two groups (p<0.05). Group 2 exhibited more pronounced swelling (140.34±10.11 mm) than group 1 (127.50±13.29 mm), with a p-value of 0.006. Pain evaluation on the second day revealed that group 2 experienced notably higher levels of discomfort (3.60±0.99) than group 1 (3.33±0.98), with a p-value of 0.0463. While there was no significant variation in mouth opening before the operation (p=0.288), the second postoperative day showed a marked difference. Group 1 demonstrated a significantly less reduction in mouth opening (45.69±6.06 mm) than group 2 (38.69±4.40 mm), with a p-value of 0.001.

Conclusion: The study has shown that administering dexamethasone before surgery led to better management of swelling, pain, and mouth opening after the procedure, compared to giving it postoperatively. These results supported the current clinical practice of utilizing corticosteroids prior to surgery to enhance patient outcomes following third molar extraction.

## Introduction

Impacted teeth, notably the third molars, constitute a prevalent dental anomaly, with global prevalence estimates ranging from 20% to 68% [1]. Surgical extraction is recognized as the most efficacious intervention for impacted teeth, with impaction of the third molars frequently serving as a primary indication for the procedure. Surgical extraction of impacted teeth is often associated with postoperative manifestations, including pain, swelling, and trismus, resulting from trauma incurred during surgical intervention [2]. Conventional strategies for alleviating these postoperative symptoms include administration of analgesics (such as ibuprofen or acetaminophen), antibiotics, and corticosteroids [3]. Among these therapeutic options, corticosteroids, particularly dexamethasone, have been identified as the most potent agents for symptom alleviation in both the preoperative and postoperative phases [4].

Dexamethasone, a synthetic glucocorticoid, is extensively used in clinical settings owing to its anti-inflammatory and immunosuppressive effects [5]. As a corticosteroid, it binds to glucocorticoid receptors, influencing the transcription of anti-inflammatory proteins and inhibiting the expression of pro-inflammatory cytokines. This action inhibits phospholipase A2, an enzyme that releases arachidonic acid, a precursor of inflammatory mediators, such as prostaglandins and leukotrienes. Consequently, dexamethasone effectively mitigates inflammation, edema, and immune activation, which is beneficial for managing postoperative complications, such as pain, swelling, and trismus [4,6].

The preoperative administration of dexamethasone frequently demonstrates greater efficacy than its postoperative counterpart, attributable to its capacity to exert effects prior to the initiation of the inflammatory cascade [7]. The administration of dexamethasone prior to surgery ensures that the pharmacological agent is available within the tissues prior to the occurrence of surgical trauma, thereby preventing the emergence of inflammation rather than solely mitigating it post-initiation [8]. This anticipatory strategy has the potential to markedly diminish postoperative discomfort, edema, and reduction in mouth opening.

Dexamethasone is superior to other anti-inflammatory drugs owing to its long half-life and potent effects, particularly in oral surgeries [9]. Preoperative administration of dexamethasone reduces pain and swelling and accelerates recovery by decreasing post-surgical complications. Its efficacy in controlling inflammation makes it the preferred choice for managing the symptoms of impaction surgeries, distinguishing it from alternative medications [8]. This study aimed to evaluate the efficacy of an 8 mg dose of dexamethasone administered pre- and postoperatively in reducing postoperative pain, swelling, and mouth opening following the surgical extraction of impacted third molars, providing insights into its optimal use in managing impaction surgeries.

## Materials and methods

Study design

This observational, prospective cohort study was conducted over a period of nine months (February 1, 2023, to October 31, 2023) in the Department of Oral and Maxillofacial Surgery at Maharaja Ganga Singh Dental College and Research Centre, Sri Ganganagar, India. This study was approved by the institution's Institutional Ethical Committee (approval number: MGSDC/SY/22/34). Written informed consent was obtained from all the patients. This study was conducted in accordance with the guidelines of the Declaration of Helsinki (2007). Patients were observed based on whether they received dexamethasone before or after surgery as part of their routine care. No randomization or intervention was performed by the researchers.

Sample size estimation

The sample size was determined in accordance with a previous study [8] that examined the effects of dexamethasone on postoperative symptoms. Utilizing a statistical power of 80% and an alpha error rate of 5%, the calculated sample size was 30 individuals in each cohort, culminating in a cumulative total of 60 participants. The sample size was estimated using the G*Power Version 3.6.9 (Heinrich-Heine-Universität Düsseldorf, Düsseldorf, Germany).

Inclusion and exclusion criteria

The investigation included systemically healthy individuals aged 18-40 years who were deemed appropriate candidates for the surgical removal of impacted third molars and were classified under the American Society of Anesthesiologists (ASA) classification system as either class I or II and patients who had already been administered dexamethasone either pre- or postoperatively based on the surgeon's decision for surgical third molar extraction. Individuals were excluded from the study if they had a documented history of corticosteroid administration within the preceding six months; exhibited known allergic reactions; had hypersensitivity to dexamethasone or any other corticosteroids; were pregnant or lactating; had systemic health conditions such as diabetes mellitus, immunosuppression, or poorly controlled hypertension; and were receiving anticoagulant or antiplatelet pharmacotherapy.

Methodology

The cohort of 60 patients was stratified into two distinct groups contingent upon the timing of dexamethasone administration. Group 1 (n=30) comprised patients who were administered an 8 mg dose of dexamethasone intramuscularly one hour before the surgical phase preoperatively, whereas group 2 (n=30) included those who were administered the same dosage immediately after the surgery in the postoperative phase. The variables of interest, including pain, swelling, and mouth opening, were assessed postoperatively on the second and seventh days.

**Figure 1:**
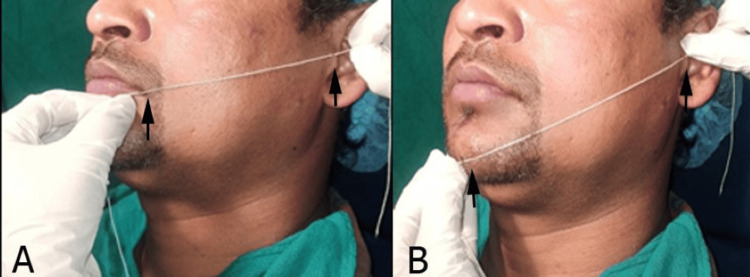
Assessment of swelling: (A) distance from the tragus of the ear to the corner of the mouth and (B) distance from the tragus of the ear to the pogonion.

The swelling was assessed by taking standardized facial measurements in three planes using a modified measuring tape method as described by Gabka and Matsumura [10]. The three facial planes measured in mm were as follows: M1, distance from the tragus of the ear to the corner of the mouth; M2, distance from the tragus of the ear to the pogonion; and M3, distance from the lateral canthus of the eye to the angle of the mandible (Figure 1).

**Figure 2:**
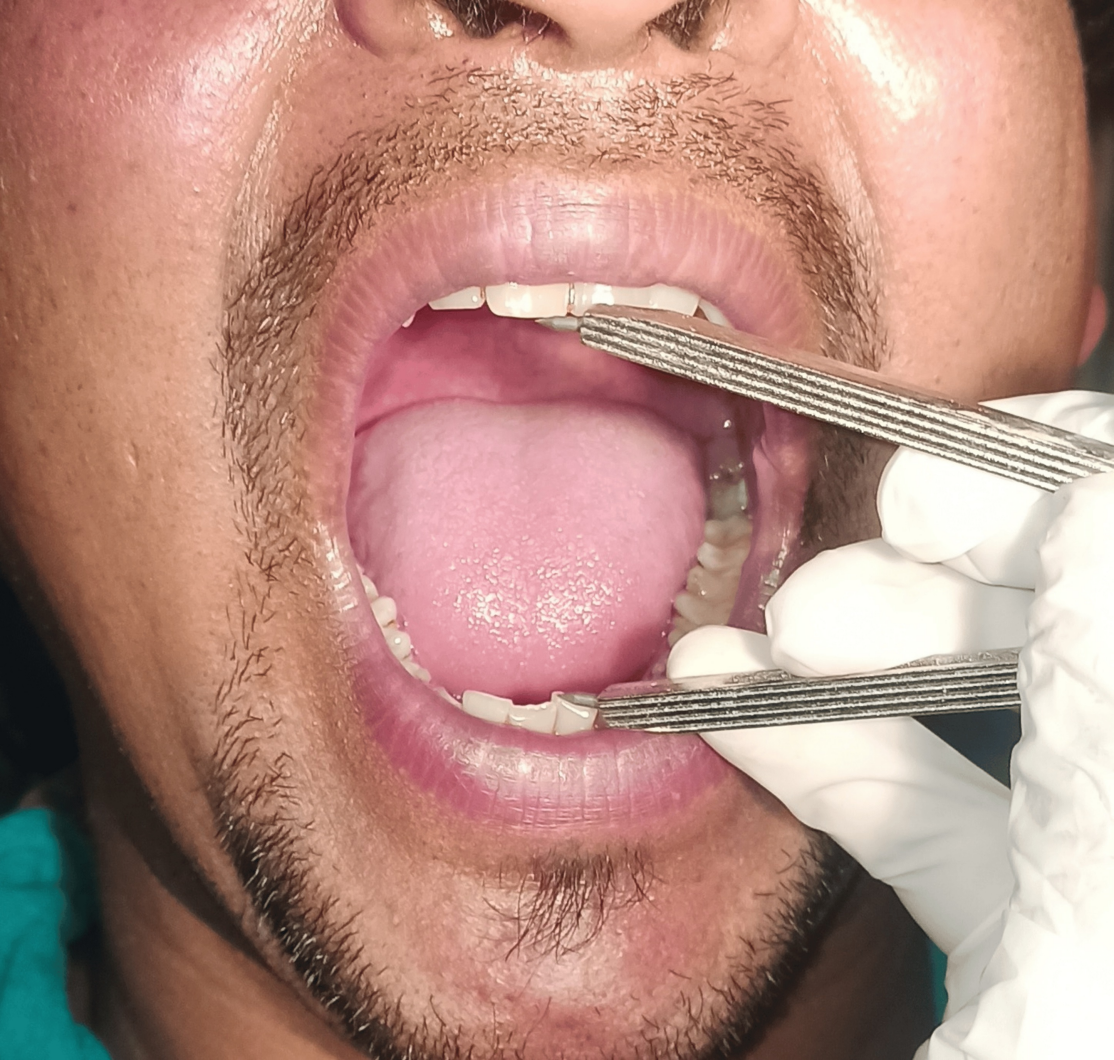
Assessment of mouth opening.

Mouth opening was evaluated by measuring the maximum interincisal opening preoperatively and on the second and seventh postoperative days using a divider and scale (Figure 2).

Pain intensity was analyzed using a Visual Analog Scale (VAS), with pain scored from 0 to 10, where 0 represented no pain and 10 indicated severe pain [11].

Statistical analysis

Data were analyzed using IBM SPSS Statistics for Windows, Version 22.0 (Released 2013; IBM Corp., Armonk, New York, United States). Descriptive statistics, such as mean and standard deviation (SD), were used to summarize continuous variables. The sex differences were evaluated using the chi-squared test. Comparisons of pain, swelling, and mouth opening between the preoperative and postoperative dexamethasone groups were performed using independent t-tests for normally distributed data. The variables between intervals were compared using repeated measures analysis of variance (ANOVA). Differences were considered statistically significant at p≤0.05.

## Results

The sex distribution between group 1 and group 2 was compared, and the results showed no statistically significant difference. In group 1, 17 (56.67%) participants were males and 13 (43.33%) participants were females. In group 2, 14 (44.67%) participants were males and 16 (53.33%) participants were females. The p-value for the difference in sex distribution was 0.438, indicating that the variation in sex proportions between the two groups was not statistically significant (p>0.05) (Table 1). 

**Table 1:** Comparison of sex distribution between the study groups using the chi-squared test. Data is presented in the form of n (%). Group 1: dexamethasone injected before the surgical procedure of third molar impaction. Group 2: dexamethasone injected after the surgical procedure of third molar impaction.

Variable	Category	Group 1 (n%)	Group 2 (n%)	P-value
Sex	Male	17 (56.67%)	14 (44.67%)	0.438
	Female	13 (43.33%)	16 (53.33%)	

The results of the study showed no significant difference in the mean age between group 1 (31.53±5.05 years) and group 2 (29.00±4.15 years) (p=0.145). There was no significant difference in preoperative swelling between the two groups (p=0.166). However, on the second postoperative day, group 2 had significantly more swelling (140.34±10.11 mm) than group 1 (127.50±13.29 mm) (p=0.006). By the seventh day, swelling remained significantly higher in group 2 (135.76±10.93 mm) than in group 1 (123.76±14.58 mm) (p=0.017). For pain assessment, no significant difference was observed preoperatively between the two groups (p=0.850). However, on the second postoperative day, group 2 reported significantly higher pain (3.60±0.99) than group 1 (3.33±0.98) (p=0.0463). By the seventh day, pain levels were similar in both groups (p=0.563). The mouth opening was not significantly different preoperatively (p=0.288). However, on the second postoperative day, group 1 had significantly greater mouth opening (45.69±6.06 mm) than group 2 (38.69±4.40 mm) (p=0.001). This trend persisted on the seventh day, with group 2 exhibiting reduced mouth opening (43.36±12.76 mm) compared to group 1 (50.00±5.50 mm) (p=0.053), as shown in Table 2.

**Table 2:** Comparison of parameters between group 1 and group 2 using the independent t-test. *p<0.05: significant. Data is presented in the form of mean±SD. Pain assessment by VAS. Group 1: dexamethasone injected before the surgical procedure of third molar impaction. Group 2: dexamethasone injected after the surgical procedure of third molar impaction. SD: standard deviation; VAS: Visual Analog Scale

Parameters	Interval	Group 1 (n=30)	Group 2 (n=30)	P-value
		Mean±SD	Mean±SD	
Age	Years	31.53±5.05	29.00±4.15	0.145
Swelling (mm)	Preoperative	123.16±14.94	129.75±9.89	0.166
	Postoperative on the second day	127.50±13.29	140.34±10.11	0.006*
	Postoperative on the seventh day	123.76±14.58	135.76±10.93	0.017*
Pain	Preoperative	5.47±1.13	5.53±0.74	0.850
	Postoperative on the second day	3.33±0.98	3.60±0.99	0.463*
	Postoperative on the seventh day	2.33±1.17	2.53±`0.64	0.563
Mouth opening (mm)	Preoperative	50.42±6.63	52.78±5.21	0.288
	Postoperative on the second day	45.69±6.06	38.69±4.40	0.001*
	Postoperative on the seventh day	50.00±5.50	43.36±12.76	0.053

Comparison of swelling, pain, and mouth opening across the preoperative, second-day, and seventh-day intervals in the two groups showed that group 1 showed significant reductions in pain (p=0.001, ƞ=0.77) and mouth opening (p=0.04, ƞ=0.21), while changes in swelling were not statistically significant (p=0.07). Group 2 demonstrated significant changes in all parameters, with pain (p=0.002, ƞ=0.88) and mouth opening (p=0.001, ƞ=0.71) decreasing significantly and swelling showing notable fluctuations (p=0.001, ƞ=0.42). In both groups, swelling was noticed on the second day after the removal of impacted third molars, and it decreased thereafter, returning to baseline values in group 1 (Table 3).

**Table 3:** Comparison of parameters between the intervals using the repeated measures ANOVA test. *p<0.05: significant. Data is presented in the form of mean±SD. Pain assessment by VAS. Group 1: dexamethasone injected before the surgical procedure of third molar impaction. Group 2: dexamethasone injected after the surgical procedure of third molar impaction. ANOVA: analysis of variance; SD: standard deviation; VAS: Visual Analog Scale

Group	Parameters	Preoperative	Postoperative on the second day	Postoperative on the seventh day	P-value	Effect size (ƞ)
Group 1 (n=30)	Swelling (mm)	123.16±14.94	127.50±13.29	123.76±14.58	0.07	0.17
	Pain	5.47±1.13	3.60±0.99	2.33±1.18	0.001*	0.77
	Mouth opening (mm)	50.78±6.64	45.69±6.06	43.37±12.76	0.04*	0.21
Group 2 (n=30)	Swelling (mm)	129.75±9.89	140.34±10.11	135.76±10.93	0.001*	0.42
	Pain	5.53±0.74	3.33±0.98	2.53±0.64	0.002*	0.88
	Mouth opening (mm)	52.79±5.22	38.85±4.40	50.59±5.41	0.001*	0.71

## Discussion

The results of this study highlighted the impact of dexamethasone administration, whether preoperatively or postoperatively, on postoperative swelling, pain, and mouth opening following third molar extraction. The study found that preoperative dexamethasone administration was more effective in controlling postoperative complications than postoperative administration. These findings align with those of previous studies, which consistently support the role of corticosteroids in reducing inflammation and improving patient recovery after oral surgery [3,4].

Swelling, a major concern following third molar extractions, was significantly lower in group 1 compared to group 2 on both the second and seventh postoperative days. The significant reduction in swelling in group 1 on the second (p=0.015) and seventh days (p=0.017) was consistent with prior studies that demonstrated the anti-inflammatory benefits of preoperative dexamethasone. According to Ustun et al., preoperative dexamethasone administration reduces the inflammatory response, leading to less postoperative edema [12]. Similarly, Sortino and Cicciù noted that dexamethasone administered preoperatively inhibited pro-inflammatory cytokines and enzymes, leading to reduced fluid accumulation and edema [2]. In comparison, patients in group 2, who received dexamethasone postoperatively, experienced delayed and less effective control of swelling, a trend consistent with prior literature suggesting that postoperative administration may not inhibit the early inflammatory cascade as effectively [4].

Pain management is another critical factor in postoperative recovery. In this study, preoperative dexamethasone administration provided better pain control, particularly on the second postoperative day, with a statistically significant difference between the groups. Previous studies, including those by Tiwana et al., have reported similar findings, where preoperative corticosteroids provided superior analgesic effects compared to postoperative administration [13,14]. Grossi et al. also found that the administration of corticosteroids before surgery significantly reduced postoperative pain owing to their ability to prevent the release of pro-inflammatory mediators [15]. The reduction in pain in both groups by the seventh day suggested that while postoperative dexamethasone eventually reduced pain, its effect was slower and less immediate than that of preoperative administration. This delayed effect is consistent with the literature, indicating that postoperative corticosteroid use may not be as effective in dampening the initial inflammatory response that causes pain [7,16].

Reduction in mouth opening, a common complication following third molar surgery, was also significantly different between the groups. On the second postoperative day, group 1 showed significantly less reduction in mouth opening than group 2, and this difference persisted on the seventh day. This outcome is supported by findings from Markiewicz et al., who showed that preoperative corticosteroid administration helps reduce muscle inflammation and fibrosis, leading to better postoperative mouth opening [17]. In contrast, patients in group 2 experienced a more prolonged reduction in mouth opening, which is likely due to the delayed onset of the effects of dexamethasone when administered after the onset of inflammation.

Intragroup comparisons of the parameters over time also provided insights into the trajectory of recovery. In group 1, swelling remained stable between the second and seventh days, and pain and mouth opening significantly decreased, suggesting that preoperative dexamethasone provided early and consistent control over swelling and pain, with stable recovery over time [11]. In group 2, although swelling decreased from the second to the seventh days, the change was not statistically significant, indicating that postoperative dexamethasone provided slower relief. However, the pain was significantly reduced, suggesting a delayed yet eventual effective analgesic effect. The reduction in mouth opening was significant, further supporting the notion that pre- or postoperative dexamethasone is not very effective at reducing muscle stiffness or improving mouth opening [12,17].

Limitations

The major limitation of the present study was the small sample size, with only 30 patients in each group, which may limit the generalizability of the findings. A larger cohort would provide more robust results and allow for subgroup analysis. Second, while the study compared the effects of preoperative and postoperative dexamethasone administration, the absence of a placebo group limited its ability to isolate the true effect of dexamethasone from other confounding factors. Additionally, the study did not account for variations in surgical technique, surgeon experience, or intraoperative complications, which could influence the postoperative outcomes. Intra-oral findings such as healing of the extraction site were not examined. Finally, the study's follow-up period was limited to seven days, and long-term outcomes, such as delayed healing or infection rates, were not assessed. Future studies should consider a longer follow-up period to evaluate the full spectrum of postoperative recovery.

## Conclusions

This study demonstrated that preoperative administration of dexamethasone resulted in superior control of postoperative swelling, pain, and trismus compared to postoperative administration. However, dexamethasone administration did not prevent a reduction in mouth opening in either group. These findings reinforce the clinical practice of using preoperative corticosteroids to optimize patient outcomes following third molar extraction to reduce postoperative pain and swelling. Further studies are warranted to examine intra-oral tissue changes occurring at the site of operation. 
